# The Third Sector in Integrated Care: Partner, Provider, or Both?

**DOI:** 10.5334/ijic.8149

**Published:** 2024-07-22

**Authors:** Michelle LA Nelson, Marianne Saragosa, Robin Miller

**Affiliations:** 1 Science of Care Institute, Sinai Health, CA; 2 Lunenfeld-Tanenbaum Research Institute, Sinai Health, CA; 3 Institute of Health Policy, Management and Evaluation, University of Toronto, CA; 4 Department of Social Work & Social Care, University of Birmingham, UK

**Keywords:** Third Sector, Cross Sector, Partnerships, Community, Community Assets, Volunteers

## Abstract

Third-sector organizations (TSOs) are recognized for having a unique and essential role in designing and delivering community-centred, sustainable health and well-being services. A World Café workshop at the 2023 International Conference on Integrated Care to explore perspectives on the questions explored the question: How do we characterize the role of the Third Sector in Integrated Care Systems? Are they Partners, Service Providers, Both or Neither? Attendees from Canada, England, Scotland, Wales, Ireland, Belgium, Denmark, and the Netherlands shared perspectives regarding facilitators and barriers to engaging TSOs in integrated care systems, drawing on experiences and practices from their communities and health systems. Building from participant perspectives, we posit that while cross-sectoral alliances between government and voluntary organizations are possible, and this engagement can contribute substantial health-promoting value to society, much work remains to be done. Meaningful collaboration requires attitudinal shifts, new working methods, rebalancing power within the relationships, and sufficient resources to support the collaboration. Creative approaches to facilitating positive engagement of TSOs within integrated care systems can address long-standing barriers and misunderstandings. Sharing and learning through research, evaluations, and networks is essential to achieve integrated care systems based on trust and committed collaboration.

## Background

The International Foundation for Integrated Care’s Call to Action in 2013 centered around the 9 Pillars of Integrated Care, stating, “Let’s be bold and strive to build alliances between communities, businesses, local government and health and care services… this is the right time to build bridges over the traditional fault lines between professions and sectors and to shift power toward community-led health and place-based approaches to integrated care.” Community organizations, or Third Sector Organizations (TSOs) are recognized for having a unique position and essential contribution in designing and delivering citizen-centred, sustainable health and well-being services [1].

TSOs can play an important role in developing and adapting services to their local context, representing and engaging underserved communities, addressing gaps in support experienced by their community, and involving volunteers in capacity building [2]. This recognition has fostered an extensive increase in the service delivery role of TSOs to the degree that such organizations have become significant providers of public health and health and care service delivery in many countries [3,4,5,6]. Collaborations with TSOs may also address resource constraints within health and care systems and support achieving the quadruple aim (2). Despite the active participation of third-sector organizations in providing health and social programs, we believe the role and contributions of these organizations as integrators of care to be insufficiently recognised and understood. This perspective shares the view of the authors and participants in an international workshop considering the contribution of TSOs to integrated care.

## International perspectives on TSO participation within integrated care systems

Participants at the World Café workshop at the 2023 International Conference on Integrated Care explored perspectives on the overarching question: *How do we characterize the role of the Third Sector in Integrated Care Systems? Are they Partners, Service Providers, Both or Neither?* Within the moderated world café session, participants from Canada, England, Scotland, Wales, Ireland, Belgium, Denmark, and the Netherlands shared experiences and perspectives regarding facilitators and barriers to engaging TSOs in integrated care systems. The discussion was organized around two predominant questions: i) What role do TSOs currently play in your integrated care systems or services? ii) What are some facilitators and barriers to engaging with TSOs, and what examples can you share with us?

Third-sector organizations fill varied roles – strategic partner, service provider, point of connection to the community, or liaison between health care and social systems. How or when TSOs fill these roles may depend on the organization’s size, mandate, financial relationships with the government, or the extent to which they share clients with health and care service organizations. Larger organizations may balance these roles simultaneously, but smaller organisations must be selective due to their capacity limitations. How a TSO participates reflects its organizational philosophy – i.e. advancing new business models, ensuring organizational sustainability, or giving voice to the communities they serve and ensuring these are represented in integrated care policies.

Organizations might join an integrated care system through ‘strategic engagement,’ where they are recognized to have a particular skill, can reach a specific population segment, or are uniquely positioned to help solve a health and care system problem. Other times, this engagement may be more performative; the TSO is invited to publicly demonstrate commitment to intersectoral collaboration, with the TSO having no meaningful influence. Transitioning between the roles of provider or partner for TSOs can be a complex process requiring all collaborators’ focused attention. Regardless of how the organizations come together, moving to authentic and meaningful intersectoral collaboration will likely require pivoting the philosophical stance of the broader, perhaps more institutional, integrated care strategic team, which is often very challenging.

A noted need for consistent terminology regarding the sector (e.g., TSO, community, volunteer, social enterprise, etc.) was identified. The variety of terms reflects the diverse composition of organizations that are not part of the public or private sectors. It makes it difficult to determine the scope of potential partners in the field. Given highly variable organizational structures, leadership models and modes of service delivery (e.g., commissioned services, social enterprise, peer associations), the Third Sector is not a sector but instead used to distinguish them from public and private entities.

Challenges and facilitators related to engagement span philosophical differences and operational issues. Concerns regarding TSO capacity to engage with numerous governance forums and fulfil the bureaucratic assurance processes required by public sector funders were raised. Integrated care system leaders highlighted challenges in collaborating with TSO. Some TSOs remained loyal to their population of focus, which made gaining agreement about partnership priorities challenging. In other cases, selected TSOs facilitate the engagement of other TSOs, and funders meet with the lead TSO to advance local initiatives. In Belgium, the public sector wanted smaller organisations to merge, making it easier to communicate with them. However, the TSOs resisted; volunteers had allegiance to ‘their’ organisation’s cause and mandate, and subsequently, the organizations remained independent but often disconnected from public systems.

Funding, whether transfer payments from publicly funded health systems or grants and donors, often supports TSOs to deliver services that benefit clients and the broader community, and it is challenging to convince public leaders to reallocate funds away from ‘core’ health and care activities. Lack of sustainable funding makes it difficult for organizations to commit to long-term initiatives, particularly those requiring them to embed new working methods. Contracts may dictate what the TSO does (or doesn’t do); in some cases, TSOs were reported to ‘become like sunflowers, reorienting to sunshine’ (orienting themselves to fundable programs and endeavours). TSOs vary in their interest in being funded by the public sector, and often, small ones do not want to be constrained by contractual agreements. In contrast, larger ones see this as an opportunity to secure longer-term funding for their workforce and services. Notably, Danish participants described a process whereby citizens could direct their personal health budget, noting that many used this funding to purchase support from TSOs, which may signal a unique position within the service delivery market.

## Discussion

Third Sector Organizations are uniquely positioned within the service delivery landscape; they are situated in the communities they serve, have a deep understanding of members’ needs, and are often seen to be in a trusted position to help [7]. Partnerships with and between TSOs are one way to expand the breadth and quality of health and social services [8,9,10]. It has been suggested that the public sector needs these partnerships as the knowledge, skill, and innovative approaches make TSOs indispensable to running a welfare state [11]. Consistent engagement, however, requires a clear understanding and operationalization of the Third Sector. The nature of TSOs contributions to society is often overlooked or discussed confusedly; within integrated care environments TSOs may be a provider of commissioned social care services, a collection of organizations predominantly focused on social determinants of health or agencies working actively in services adjacent to local health care systems such as policy, advocacy or research organizations. The lack of clarity and variation surrounding the voluntary sector organizations’ definitions, terminology, and classification [12] has resulted in the collective being described as a ‘loose and baggy monster’ [13]. Confusion surrounding the role and scope of TSOs contributions may add to health and social care professionals’ skepticism of their value. For example, while innovative programming offered by TSOs is recognized as strengthening their ability to meet community needs nimbly [14], it may also hinder measurement and reporting that would appeal to funders and other interested parties [15,16]. However, implementing a more tightly regulated model could help to increase TSO involvement in integrated care. Still, care must be taken to ensure that increased visibility does not result in TSOs replacing publicly funded services rather than complementing them. With proper regulatory control, this can be a mechanism for protecting TSO from potential exploitation [12].

TSOs often have complementary and secondary missions to other sectors, with corresponding short-term funding models [16], which compound the frustration and contribute to the sectors’ continuous precarity. Perceived competition between service providers for funding may hinder TSOs in accessing and maintaining sustainable financing, and these short-term funding cycles, with limited evaluation, make it difficult to convey longer-term impact to funders. However, as shifts are being made to integrating health and social care, TSOs must demonstrate their value for money and ensure quality-of-service provisions. Following Lebec and Dudau (2023), TSOs engaging in performance management through adaptive, collaborative and sustainable strategies over a longer-term horizon may support variety in public service provision.

Cross-sectoral alliances between government and voluntary organizations can contribute to public value for TSOs and opportunities for collaborative problem-solving, decision-making, and even tackling socioeconomic disadvantages [17]. This is important for TSOs, as collaboration leads to expanding networks and other service delivery options. Building effective and sustainable collaborations between TSOs and other actors requires a collaborative governance structure for partnership. According to the literature, this model is characterized as a formal process that is deliberative and consensus-oriented, aiming to manage specific programs, assets, or services to achieve a common goal [18].

## Conclusion

TSOs have always had a role in improving the health and well-being of populations and, in many ways, preceded public approaches. Meaningful engagement within integrated care is possible but requires attitudinal shifts, new working methods, rebalancing power within the relationships, and sufficient resources to support the collaboration. Creative approaches to facilitating positive engagement of TSOs within integrated care systems can address long-standing barriers and misunderstandings. Sharing and learning through research, evaluations, and networks is essential to achieve integrated care systems based on trust and committed collaboration.
